# Deep vein thrombosis due to May-Thurner syndrome: a case report

**DOI:** 10.1186/s12872-020-01515-z

**Published:** 2020-05-19

**Authors:** Yan Meng, Hao Qin, Qiang Ma, Junbo Zhang, Bo Zhang, Honggang Pang, Qian Yin, Hongyan Tian

**Affiliations:** Department of Peripheral Vascular Diseases, the First Affiliated Hospital of Xi’an Jiaotong University, No. 277 Yanta Road, Xi’an, 710061 China

**Keywords:** May-Thurner syndrome, Iliac vein plasty, Deep vein thrombosis, Endovascular management, Case report

## Abstract

**Background:**

May-Thurner syndrome (MTS) or Cockett’s syndrome is a rare clinical syndrome, which refers to the compression of the left common iliac vein (LCIV) by right common iliac artery and vertebral body. Complications of MTS include deep vein thrombus formation and even life-threatening pulmonary embolism.

**Case presentation:**

Here, we report the case of a 60-year-old female patient with a complaint of swelling in the left lower limb and pain for 5 days. Computed tomography angiography indicated MTS, and thrombus formation of left external iliac vein and femoral vein. The patient was diagnosed with deep venous thrombosis (DVT) and MTS. The patient underwent ascending venography from the lower extremity to inferior vena cava (IVC) and then to the pulmonary artery with IVC filter implantation, left iliac vein balloon plasty, and stent placement. The patient visited the hospital for the removal of IVC filter, 28 days after the operation. After the interventional therapy, the patient had no in-stent restenosis and had remission during the 2-year follow-up.

**Conclusions:**

This case presents a successful management of MTS in presence of DVT. Although clinicians are rarely aware, the presence of unilateral lower limb swelling and thrombosis may be the manifestations of MTS.

## Background

Deep venous thrombosis (DVT) is more common in the left side (60%) than in the right side of the lower extremity [1]. Previous studies have revealed that this might be due to the compression of left common iliac vein (LCIV) by the right common iliac artery (RCIA). This condition is known as May-Thurner syndrome (MTS) [2]. This compression leads to the obstruction of venous outflow and increases the risk of DVT [3]. However, MTS presented variable clinical symptoms, which ranges from unilateral lower extremity swelling without thrombosis to minor cases of DVT [4].

Patients who have lower extremity DVT are at the risk of thrombus detachment and circulating it to the lung. This is known as pulmonary embolism (PE). For patients with contraindication to anticoagulant therapy, permanent or removable filters are placed in the inferior vena cava (IVC) to prevent PE [5]. For patients with both MTS and DVT, leg pain or swelling can be improved by stent placement [5].

In this report, a successful treatment experience of MTS in presence of DVT by the use of filter insertion, balloon plasty, and stent placement was discussed.

## Case presentation

**Fig. 1:**
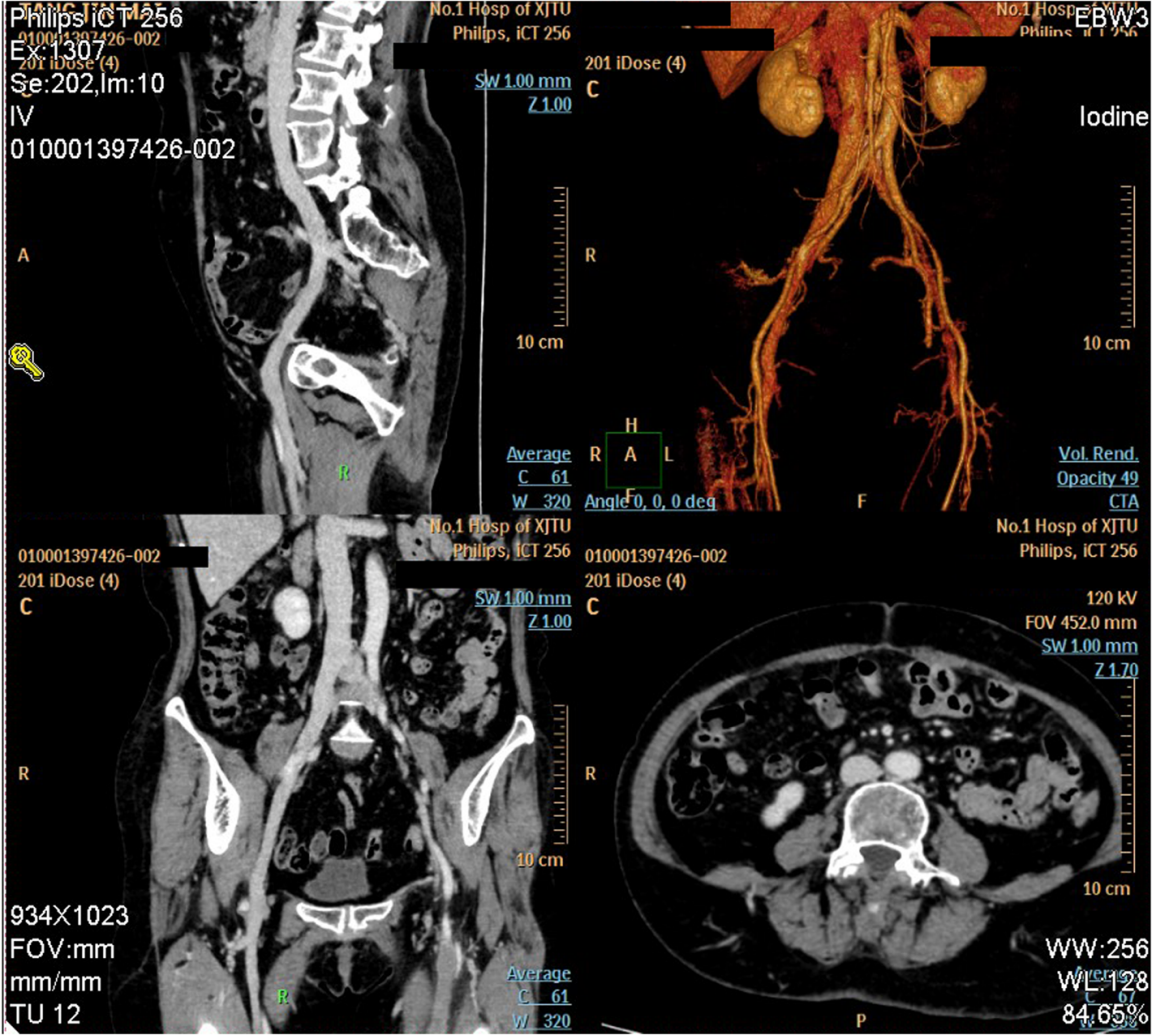
CT arterial angiography before operation

A 60-year-old female patient was hospitalized for swelling and pain in the left lower extremity for 5 days. It was reported that, 5 days ago, the patient experienced swelling of thigh, which gradually spread to the lower leg, and increased local skin tension and temperature. The patient had no fever, shortness of breath, chest pain, cough, expectoration, hemoptysis, amaurosis, or syncope; no special relevant medical, family, psycho-social past histories, and no history of chronic lower limb symptoms, even during her previous pregnancy. Deep vein ultrasound of the lower extremity at local hospital reported thrombosis of left common femoral vein, superficial femoral vein, deep femoral vein, popliteal vein, anterior tibial vein, posterior tibial vein, and intermuscular vein for the first time. She had no history of surgery or catheterization. On physical examination, her heart rate was 88 bpm, and blood pressure was 130/74 mmHg. Examination of the left lower limb revealed swelling and edema with moderate tenderness and pain, while the right lower limb was normal. Blood tests revealed normal blood routine and normal liver and kidney functions. Computed tomography angiography (CTA) indicated compression of LCIV by RCIA, narrowed LCIV lumen, thrombi formed from the femoral vein to left external iliac vein, and collateral circulation formed between bilateral external iliac vein (Fig. 1). She was diagnosed with DVT along with MTS.

**Fig. 2:**
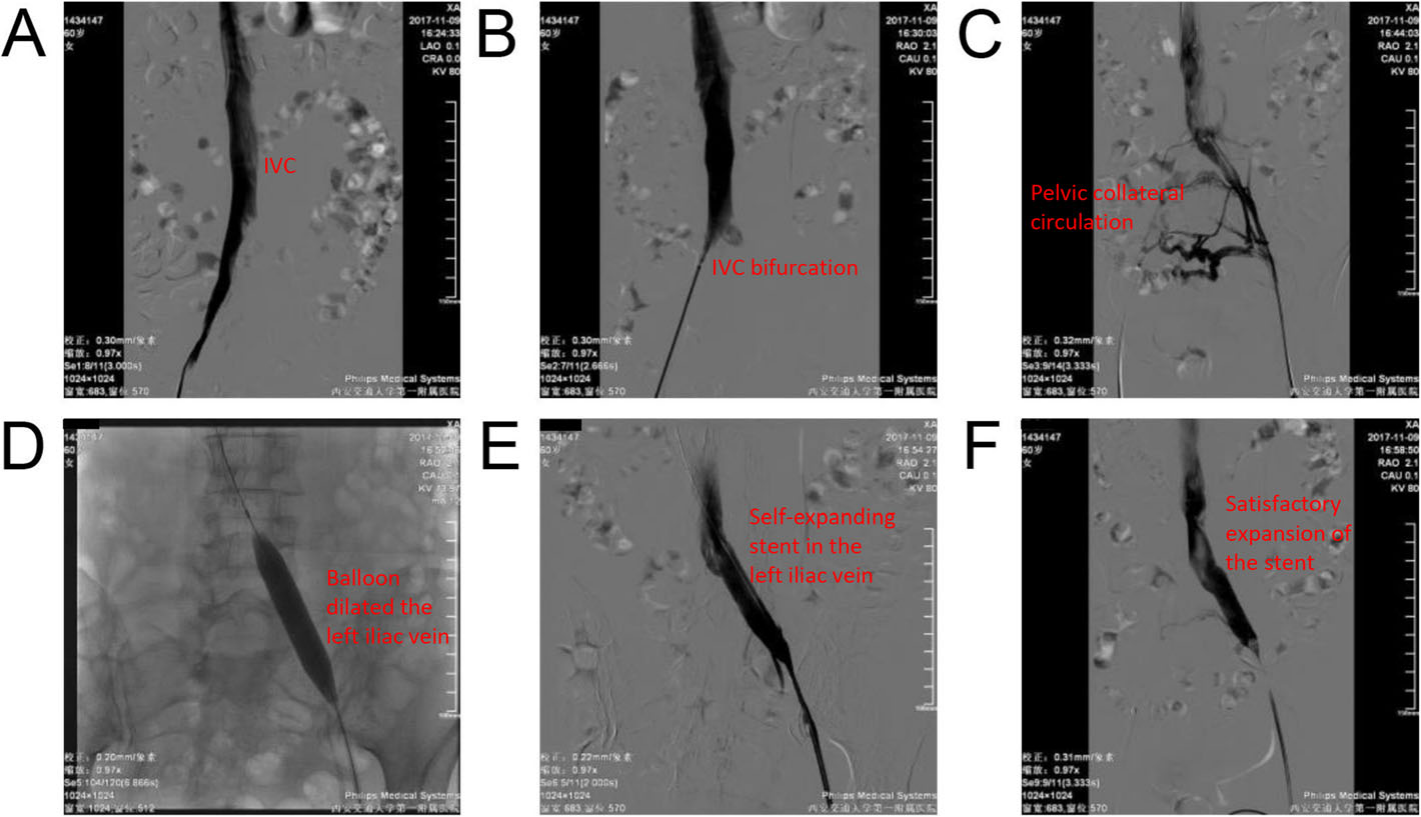
Surgical procedure of venography. **a** Inferior vena cava angiography showed smooth blood flow. **b** Insertion of a Cordis Optease vena cava filter. **c** The left iliac vein was severely narrowed and the pelvic collateral circulation remained open. **d** 14 mm–60 mm balloon dilatation of left iliac vein. **e** An E-lunimexx 14 mm–60 mm self-expanding stent was implanted into the left iliac vein. **f** The stent was in good position, and the expansion remained satisfactory

The patient underwent IVC angiography, filter placement, left iliac vein plasty, and stent implantation under local anesthesia. The right femoral vein was punctured successfully with Seldinger technique [6] and then placed into the catheter sheath. Contrast agent was passed smoothly through IVC without filling defect (Fig. 2a). The sheath of the filter was transported, and a Cordis Optease filter was placed in the IVC at second lumbar vertebrae (Fig. 2b), followed by puncturing of the left femoral vein. The catheter sheath was then inserted; angiography revealed severe stenosis of the left external iliac vein and the common iliac vein, and the pelvic collateral compensation was seen through contralateral reflux (Fig. 2c). A 14 mm - 60 mm balloon was inserted into the IVC through a narrow segment of left external iliac vein and common iliac vein (Fig. 2d). Then, an E-lunimexx 14 mm - 60 mm self-expanding stent was inserted (Fig. 2e). The stent was in good position, and the expansion remained satisfactory (Fig. 2f). The contrast medium was passed through smoothly, and the original stenosis was relieved. At the end of the operation, the sheath of the bilateral inguinal area was removed, and the wound was dressed with a bandage. The patient returned to the ward safely and tolerated well, and was discharged 5 days after the surgery. The patient was prescribed with oral rivaroxaban and aspirin. After 28 days of the surgery, she came back to the hospital for the removal of IVC filter. This procedure was successful. The DVTs were ablated after 4 months of anticoagulation therapy.

## Discussion and conclusions

May-Thurner syndrome was initially reported in 1956 by May and Thurner. They found that 22% of the studied adult subjects had thickened left iliofemoral venous wall, where the RCIA has crossed the LCIV. The obstruction was termed as a “spur,” and this was equal to a callus formation under chronic repetitive stimulation from the compression of LCIV, by pulsating RCIA. Proliferation of cells at LCIV is the sequencing event, this might in turn aggravate stenosis and trigger venous thrombosis [3, 7]. Previous studies have revealed that the incidence of LCIV compression ranged 14–32% [8]; however, 2–5% of these patients complained of lower extremity symptoms [9]. MTS is more commonly observed in female patients than in male patients, and 18–49% of the MTS cases had left lower extremity DVT [10–12]. Moreover, pregnancy is a risk factor for DVT formation [13].

The diagnosis of MTS relies on imaging examinations. Certain tests should be conducted in patients with unilateral leg swelling to exclude MTS. Ultrasound could identify thrombus formation of DVT. However, in most of the patients, deep veins in the pelvis cannot be fully visualized by ultrasound [4]. If MTS is suspected with characteristic ultrasonography, further examination should be conducted by computed tomography venography (CTV) or magnetic resonance venography (MRV). Computed tomography venography and MRV are relatively noninvasive methods, which are used to visualize the deep veins in the pelvis. These methods can reveal the overlying of RCIA on LCIV, and the presence of compression and stenosis [4, 14]. Another technique involves the combination of contrast venography and intravascular ultrasound, which is considered as a reliable approach for identifying abnormal venous blood flow and precisely locate the etiology and the degree of compression [4].

The traditional therapy for MTS is surgery and is outdated. The precise optimal treatment strategy remains to be determined. Endovascular treatments might be the first choice for effective management of MTS, which include techniques such as catheter-directed thrombolysis, IVC filter implantation, catheter-directed balloon angioplasty, and stenting placement [5, 13, 15]. Selective catheter-directed thrombolysis is considered effective for thrombus clearance and alleviation of symptoms [13]. IVC filter, which is also called as Greenfield filters, can efficiently prevent the spread of ruptured thrombus to the lungs [5]. Balloon angioplasty is often used in combination with stent placement and directly provides relief from anatomic stenosis of LCIV by the compression of RCIA [5]. According to a research in 39 patients with MTS, endovascular treatment showed 87% success rate [11].

In our study, the patient had acute swelling and pain in her left lower limb for 5 days. Computed tomography angiography showed DVT formation on the left external iliac vein and femoral veins, and obvious stenosis of LCIV due to the compression of RCIA. The patient underwent IVC filter placement, left iliac vein plasty, and stent implantation. The swelling of her left leg was largely alleviated after the operation, and she was in good condition without chronic symptoms of MTS for a two-year follow-up period. In MTS patients with only 6 months of warfarin antithrombotic therapy, the reported 12-month stent patency rate was 78%, while for the patients with more than 6 months of systemic anticoagulation or dual-antiplatelet therapy, the same was 89% [16]. At the time of discharge from the hospital, the patient had residual thrombi and was suggested to take rivaroxaban 10 mg and aspirin 100 mg once a day as long term anti-coagulation strategy, and to monitor coagulation function test, every 3 month in outpatient department.

Clinicians should be aware of the signs and symptoms of MTS and should consider this diagnosis in cases where the patients are presented with unexplained lower limb swelling or women with extensive left lower limb DVT during pregnancy.

Also, endovascular treatment appears to be a reliable therapy of MTS and can effectively alleviate the symptoms in patients.

## Data Availability

All data generated or analysed during this study are included in this published article.

## Abbreviations

CTA: Computed tomography angiography
CTV: Computed tomography venography
MRV: Magnetic resonance venography
DVT: Deep vein thrombosis
MTS: May-Thurner syndrome
LCIV: Left common iliac vein
RCIA: Right common iliac artery
PE: Pulmonary embolism
IVC: Inferior vena cava
